# Supplementary material for: One-Year Progression and Risk Factors for the Development of Chronic Kidney Disease in Septic Shock Patients with Acute Kidney Injury: A Single-Centre Retrospective Cohort Study
Source: J Clin Med. 2018 Dec 15;7(12):554. doi: 10.3390/jcm7120554 (PMC6306914; doi:10.3390/jcm7120554)
Supplement: Supplementary file 1 [file jcm-07-00554-s001.pdf]

Supplementary 1. Univariate logistic regression of factors associated with the occurrence of CKD after 1 year including follow-up loss patients (sensitivity analysis).

Discharge Creatine level	Univariate analysis		
	OR	95% CI	*p*
All CKD	1.915	1.351 – 2.714	< 0.001
No CKD	1.550	1.174 – 2.047	0.002
Partial CKD (20% incidence)	1.449	1.120 – 1.874	0.005

Abbreviations: OR = odds ratio; CI = confidence interval; CKD = chronic kidney injury.
